# Differential response effects of data collection mode in a cancer screening study of unmarried women ages 40–75 years: A randomized trial

**DOI:** 10.1186/1471-2288-8-10

**Published:** 2008-02-29

**Authors:** Melissa A Clark, Michelle L Rogers, Gene F Armstrong, William Rakowski, Frederick J Kviz

**Affiliations:** 1 Center for Gerontology and Health Care Research, Program in Public Health, Brown University, Providence, Rhode Island, USA; 2 Community Health Sciences, School of Public Health, University of Illinois, Chicago, Illinois, USA

## Abstract

**Background:**

Little is known about the impact of data collection method on self-reported cancer screening behaviours, particularly among hard-to-reach populations. The purpose of this study is to examine the effects of data collection mode on response to indicators of cancer screenings by unmarried middle-aged and older women.

**Methods:**

Three survey methods were evaluated for collecting data about mammography and Papanicolaou (hereafter, Pap) testing among heterosexual and sexual minority (e.g., lesbian and bisexual) women. Women ages 40–75 were recruited from June 2003 – June 2005 in Rhode Island. They were randomly assigned to receive: Self-Administered Mailed Questionnaire [SAMQ; N = 202], Computer-Assisted Telephone Interview [CATI; N = 200], or Computer-Assisted Self-Interview [CASI; N = 197]. Logistic regression models were computed to assess survey mode differences for 13 self-reported items related to cancer screenings, adjusting for age, education, income, race, marital status, partner gender, and recruitment source.

**Results:**

Compared to women assigned to CATI, women assigned to SAMQ were less likely to report two or more years between most recent mammograms (CATI = 23.2% vs. SAMQ = 17.7%; AOR = 0.5, 95% CI = 0.3 – 0.8) and women assigned to CASI were slightly less likely to report being overdue for mammography (CATI = 16.5% vs. CASI = 11.8%; AOR = 0.5, 95% CI = 0.3 – 1.0) and Pap testing (CATI = 14.9% vs. CASI = 10.0%; AOR = 0.5, 95% CI = 0.2 – 1.0). There were no other consistent mode effects.

**Conclusion:**

Among participants in this sample, mode of data collection had little effect on the reporting of mammography and Pap testing behaviours. Other measures such as efficiency and cost-effectiveness of the mode should also be considered when determining the most appropriate form of data collection for use in monitoring indicators of cancer detection and control.

## Background

Over 18 million women aged 40–75 in the United States are currently unmarried [1]. The designation of "unmarried" refers to women who are legally separated, divorced, widowed, or never legally married. Sexual minorities (e.g., lesbians and bisexuals) are an important segment of the unmarried female population. Sexual minority women may be living in committed relationships comparable to married heterosexual couples but are unable to legally marry in any state except Massachusetts. Although few studies include sufficient samples of unmarried women for analysis, data suggest that the risk for breast and cervical cancer may be higher for subgroups of unmarried women than women in general [2-5]. Therefore, the burden of disease and risk of adverse health outcomes may be greater for unmarried women if detection is delayed or forgone.

Determining effective modes of obtaining sensitive personal information is one important aspect to improving rates of cancer detection and control among unmarried women. Prior studies have documented that the methods used to elicit information can influence both individuals' willingness to disclose personal information and the quality of data that are obtained. The advantages of anonymity in self-administered questionnaires (SAQ) relative to telephone and face-to-face interviews have been demonstrated [6-8]. However, the use of paper-and-pencil SAQ is disadvantageous in terms of: lower unit response rates, higher levels of missing data, higher numbers of inconsistent or illogical responses across related questions, and limitations on questionnaire complexity such as skip patterns [9]. Conversely, significant advantages in data quality and flexibility of questionnaire format with telephone and face-to-face interviews have been well-documented, including quality of recorded answers, control of response order, and use of complicated skip patterns [9,10].

Computer assisted self-interviewing (CASI) has advantages in data quality and questionnaire design flexibility comparable to telephone and face-to-face interviews [11-13]. However, studies comparing CASI to other modes of data collection for reporting sensitive behaviours have shown varying results. Some investigators have found mixed [12] or limited main effect differences [13-15] for CASI versus face-to-face interviews and SAQ. Others have found that CASI may be as good as, or even better than, face-to-face interviews at fostering a sense of privacy and increasing the willingness of respondents to report sensitive information [11,16-19].

Respondent age, general trust in others, attitudes about privacy and confidentiality, and attitudes towards computers may influence reactions towards CASI [15,20,21]. These issues may be particularly relevant for women 40–75 years who are age-eligible for breast and cervical cancer screening, and who may have less experience with computers than younger women. In addition, sexual minority women may be particularly concerned about privacy and confidentiality.

There is limited information about the effect of interview mode on the willingness of unmarried middle-aged and older women to reveal personal information about cancer-related attitudes and practices. Without specific information about differential effects of data collection mode, it is impossible to determine the extent to which women are under-represented in surveillance and interventions because they are less likely to participate and/or are afraid to acknowledge potentially sensitive information. In addition, we must have methods that provide optimally valid and reliable self-reported behavioural and attitudinal data. Previous studies have found that women self-report cancer screenings at rates higher than indicated in clinical records [22-26]. These issues are particularly important as researchers and health care organizations seek valid, cost-effective forms of data collection for interventions to improve quality of care indicators. Therefore, the objectives of this study were to:

1. Describe the utility and feasibility of different modes for collecting data from middle-aged and older unmarried women;

2. Examine the effects of randomized interview mode on responses to indicators of mammography and Pap test screening; and

3. Determine whether the effects of interview mode on responses to indicators of mammography and Pap test screening differ by partner gender.

## Methods

The study design included a three-step process: recruitment, allocation to eligibility strata, and randomization to data collection mode.

### Sample and recruitment

Women were eligible if they were legally unmarried, were aged 40–75 years, currently received the majority of their health care in Rhode Island, and had never been diagnosed with cancer other than non-melanoma skin cancer. Women with a previous diagnosis of cancer were excluded because the overall focus of the study was on cancer screening behaviours, and the experiences for survivors have been shown to be different than for women who have never had a cancer diagnosis [27,28].

We used principles of targeted and respondent driven sampling [29] to recruit and enroll participants. Comparable strategies were used to recruit heterosexual and sexual minority women. A total of 773 women were recruited and screened for eligibility over 25 months (June 1, 2003 – June 30, 2005). Six general sources were used for recruitment: (a) community settings (n = 146); (b) health fairs (n = 123); (c) mailings and flyers (n = 153); (d) print media (n = 135); (e) staff and participant social networks (n = 146); and other (n = 70). For additional information about participant recruitment, see Clark et al. [30].

### Allocation to eligibility strata

Upon contact with a potential participant, we administered a telephone screening protocol following informed consent. To determine eligibility and to ensure comparable marital status and sexual orientation characteristics within interview mode, women were asked their marital status, followed by the gender of a current partner or gender preference of a partner if they were not currently in a relationship. As depicted in Table 1, women were then allocated into one of six marital status/partner gender strata: (a) never married women who partner with women [WPW] or with either women or men [WPWM] (hereafter referred to as WPW); (b) previously married WPW [includes WPWM]; (c) never married women who partner with men [WPM]; (d) previously married WPM; (e) never married women with no partner preference [NPP] and (f) previously married NPP. Strata (e) and (f) included women who reported no interest in having a partner and refused to select the gender of a potential future partner. Demographic characteristics of NPP were comparable to WPM and therefore were subsequently combined with WPM for all analyses.

**Table 1:** Sample sizes of marital status by partner gender strata for study participants, Rhode Island, 2003–2005

	**Marital Status**	
	
**Partner gender**	Never Married (n)	Previously Married (n)
WPW	144	69
WPM	158	236
NPP	18	5

WPW = Women who partner with women; WPM = Women who partner with men; NPP = No partnering preference

### Randomization to data collection mode

After eligibility screening, we asked each woman for permission to be randomized to data collection mode. Each woman had an equal probability of being assigned to one of the three data collection modes: Self-Administered Mailed Questionnaire [SAMQ], Computer-Assisted Telephone Interview [CATI], and Computer-Assisted Self-Interview [CASI]. We used a systematic block randomization schedule to make mode assignments within each of the six marital status/partner gender strata to control for the long recruitment period and non-probability based sampling methods.

Women assigned to SAMQ received a 28-page booklet-form questionnaire. Women assigned to CATI completed a 35–40 minute telephone interview. Women assigned to CASI chose to complete the assessment in one of two ways: (a) laptop computer provided and monitored by research staff at locations chosen by the study participant [CASI-I]; or (b) computer disk mailed to the participant's home and returned in a self-addressed postage-paid mailer [CASI-D]. Audio technology was available for the CASI-I condition but not CASI-D. The time needed for assessment by CASI was comparable to CATI. The CATI and CASI programs were designed using the Ci3 software from Sawtooth Technologies [31].

Women in the SAMQ were asked to return the questionnaire within two weeks. Up to 10 follow-up telephone reminders were made to non-responders. Similarly, up to 10 attempts were made to collect data from women in the CATI and CASI modes.

There were two alternatives for eligible women who did not provide data by the mode to which they were randomized. First, women who were randomized who did not provide data after 10 contact attempts were considered non-respondents. Non-respondents were offered the opportunity to participate by either of the two data collection modes to which they were not assigned. Second, women who did not agree to be randomized were provided the option to self-select (self-choice) the mode of data collection. The protocol for self-choice was comparable to that for random assignment. Women in the self-choice group were included in analyses comparing those who did and did not agree to randomization. However, they were not included in analyses of the effect of interview mode on reports of cancer screening behaviours.

### Indicators of cancer screenings

We included items in the survey related to mammography and Pap test screening. We provided women with a description of the screening test prior to asking items about the exam. Five variables were related to mammography screening and were coded as dichotomous (yes/no) indicators: no mammogram in past two years, ever put off/avoided the test, two or more years between most recent exams, no plan to get the exam with the next two years, and perceived difficultly with the exam because of breast shape or size. Five parallel items were related to Pap testing: no Pap test in past three years, ever put off/avoided the test, three or more years between most recent exam, no plan to get the exam with the next three years, and perceived difficultly with the exam because of body shape or size. Consistent with current recommendations [32-34], we used screening intervals of two years for mammography and three years for Pap testing.

In addition to items specific to the tests, we included three variables related to cancer screenings more generally. One variable was a composite of four questions with comparable response options about reported barriers to cancer screening. Women were classified as reporting a barrier if they endorsed one or more of the following: problems taking time off work; transportation problems; health-related limitations; or difficulties with getting someone to care for dependents. Second, women were asked if they had ever put off or avoided cancer screenings because of embarrassment in showing their body. Finally, women were asked if they had ever changed the place for cancer screening exams because of embarrassment in showing their body.

### Analysis plan

We analyzed the data using SAS, version 9.1 [35]. Our first set of analyses was conducted to examine the utility and feasibility of different modes for collecting data from middle-aged and older unmarried women. First, we compared participant characteristics by randomly assigned mode of data collection. Second, we compared characteristics for women who agreed to be randomized versus those who chose their data collection mode [self-choice]. Third, we compared women who completed the assessment in the assigned mode to: (a) women who completed the assessment in a different mode; and (b) women who did not complete the assessment. Next, we assessed the relationship between number of contacts after randomization and response rate by assigned mode of data collection.

In the second set of analyses, we specifically examined the effects of interview mode on the responses to cancer screening indicators. For these analyses, we only included women who completed the assessment in the assigned mode. We examined distributions and computed proportions for all variables by randomization group. We then used Pearson Chi-square tests to compare the proportion of women who endorsed each of the 13 variables across data collection mode. Next, we computed odds ratios with 95% confidence intervals (CI) to assess differences between the variables reported by data collection modes, adjusting for partner gender, marital status, age, education, employment, race, and recruitment source. Finally, we tested interactions between data collection mode and partner gender.

## Results

### Sample composition

**Figure 1:**
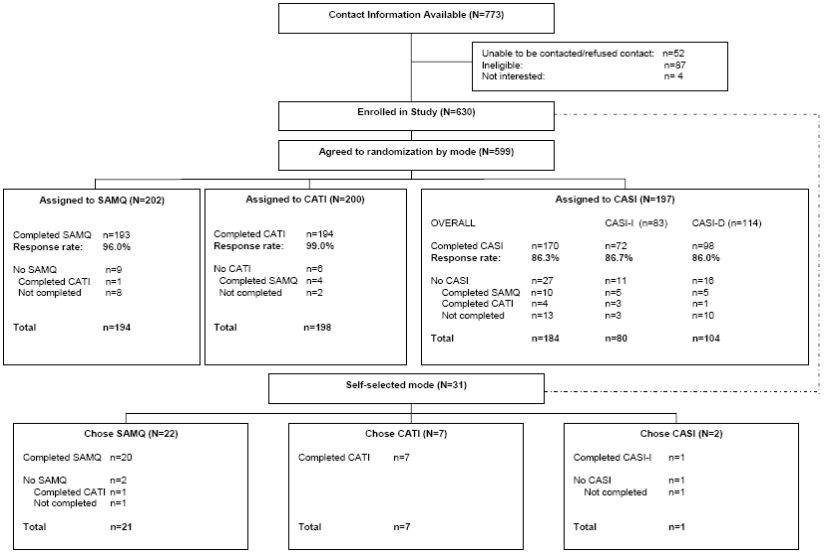
**Participant flow in the Cancer Screening Project for Women, Rhode Island, 2003–2005**. SAMQ = Self-administered mailed questionnaire. CATI = Computer-assisted telephone interview. CASI = Computer-assisted self interview.

The numbers of women in each of the marital status-partner gender strata are shown in Table 1. A total of 630 women were enrolled in the study (Figure 1). Of these, 599 women agreed to be randomized to mode of data collection. Because of unequal sample sizes in each marital status/partner gender strata, the three randomized groups were slightly different in size (SAMQ = 202; CATI = 200; CASI = 197). Among those randomized to SAMQ and CATI, nearly all completed the questionnaire in the assigned mode (96% and 99%, respectively). Only 86.3% (n = 170) of women randomized to CASI completed the interview in the assigned mode (CASI-I = 86.7% and CASI-D = 86.0%). Reasons for not completing the assessment in the assigned mode included: unable to contact after randomization (SAMQ = 2, CASI = 3), changes in personal or family circumstances (CATI = 2, SAMQ = 1, CASI = 3), limited English competency (SAMQ = 2), and lost interest in the study (SAMQ = 3, CASI = 7). Figure 1 also shows the distribution of the 31 women who refused randomization (self-choice).

**Figure 2:**
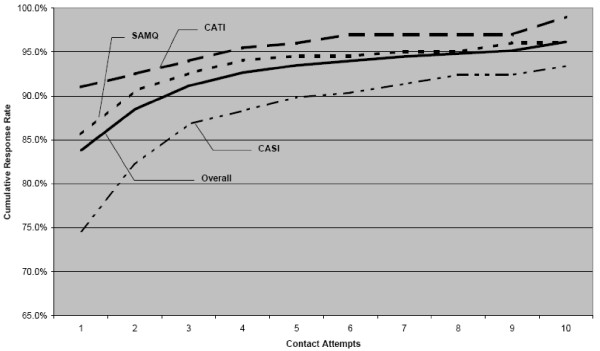
**Response rate by Contact Attempts in the Cancer Screening Project for Women, Rhode Island, 2003–2005**. SAMQ = Self-administered mailed questionnaire. CATI = Computer-assisted telephone interview. CASI = Computer-assisted self interview.

Figure 2 shows the relationship between number of contacts with participants after randomization and response rates by assigned mode. More contact attempts with participants were required for CASI relative to SAMQ and CATI to achieve comparable response rates. For example, to achieve a response rate of 90%, six contact attempts on average were required for women assigned to CASI compared to two for SAMQ and one for CATI.

### Participant characteristics by data collection mode

There were no differences in participant characteristics by randomly assigned mode of data collection (CATI vs. SAMQ vs. CASI; Table 2). Within the CASI condition, WPM/NPP were equally likely to choose CASI-I and CASI-D while almost 70% of WPW chose CASI-D. There was no substantial difference in choice of CASI condition for women without a college degree. However, the majority of women with a college degree selected CASI-D. The majority of women who were not employed and those who were non-white chose CASI-I, while employed women and white women chose CASI-D. Women recruited by print media, mailings/flyers, and personal networks were more likely to choose CASI-D, while those recruited at community settings, health fairs, or other settings were more likely to choose CASI-I.

**Table 2:** Participant characteristics by mode of data collection, Rhode Island, 2003–2005

	**Assigned Mode**						**Choice of CASI**			
	**CATI (n = 200)**		**SAMQ (n = 202)**		**CASI (n = 197)**		**CASI-I (n = 83)**		**CASI-D (n = 114)**	
**Characteristic**	**n**	**%**	**n**	**%**	**n**	**%**	**n**	**%**	**n**	**%**

Partner gender *										
WPW	64	32.0	68	34.0	68	34.0	22	32.4*	46	67.7
WPM or NPP	136	34.1	134	33.6	129	32.3	61	47.3	68	52.7
Marital status										
Never married	100	33.1	103	34.1	99	32.8	37	37.4	62	62.6
Previously married	100	33.7	99	33.3	98	33.0	46	46.9	52	53.1
Age in years										
40–49	84	33.5	90	35.9	77	30.7	25	32.5	52	67.5
50–59	63	33.3	62	32.8	64	33.9	31	48.4	33	51.6
60–69	39	32.8	39	32.8	41	34.5	20	48.8	21	51.2
70–75	14	35.0	11	27.5	15	37.5	7	46.7	8	53.3
Level of formal education*										
High school, some college, or technical training	80	33.9	77	32.6	79	33.5	42	53.2*	37	46.8
College degree or more	118	35.0	116	34.4	103	30.6	38	36.9	65	63.1
Working full-time or part-time*										
No	54	32.3	55	32.9	58	34.7	38	65.5*	20	34.5
Yes	144	35.8	136	33.8	122	30.4	40	32.8	82	67.2
Hispanic ethnicity										
Yes	6	26.1	8	34.8	9	39.1	5	55.6	4	44.4
No	192	35.2	183	33.5	171	31.3	74	43.3	97	56.7
Race*										
Black, Native American, Biracial, Multiracial	54	40.0	39	28.9	42	31.1	28	66.7*	14	33.3
White	143	33.0	152	35.1	138	31.9	52	37.7	86	62.3
Source of recruitment*										
Print media	47	36.2	45	34.6	38	29.2	11	29.0*	27	71.0
Community settings	30	30.9	38	39.2	29	29.9	17	58.6	12	41.4
Mailings/flyers	38	34.6	36	32.7	36	32.7	14	38.9	22	61.1
Personal networks	37	28.5	41	31.5	52	40.0	15	28.9	37	71.2
Health fair/other	48	36.4	42	31.8	42	31.8	26	61.9	16	38.1

SAMQ = Self-Administered Mailed Questionnaire; CATI = Computer Assisted Telephone Interview.

CASI = Computer Assisted Self Interview; CASI-I = In-person CASI; CASI-D = Mailed disk CASI.

WPW = Women who partner with women; WPM = Women who partner with men; NPP = No partnering preference.

Note: There were no significant differences across mode of data collection (CATI vs. SAMQ vs. CASI).

* Significant difference between types of CASI (in-person vs. mailed disk).

Participant characteristics by status of actual participation are shown in Table 3. Among those randomized, older women and those who worked full- or part-time were more likely to complete the assessment in the assigned mode. Women without a college degree and Hispanic women were more likely to choose the self-choice condition (Total Randomized vs. Self-Choice).

**Table 3:** Participant characteristics by status of participation (randomized vs. self-choice conditions), Rhode Island, 2003–2005

	**Randomized to Mode (Type of Participation)**									
			
	**Completed in Assigned Mode** **(n = 557)**		**Completed in Different Mode** **(n = 19)**		**Did Not Complete** **(n = 23)**		**Total Randomized** **(n = 599)**		**Self-Choice** **(n = 31)**	
	
**Participant Characteristics**	**n**	**%**	**n**	**%**	**n**	**%**	**n**	**%**	**n**	**%**
Partner gender										
WPW	190	95.0	5	2.5	5	2.5	200	93.9	13	6.1
WPM or NPP	367	92.0	14	3.5	18	4.5	399	95.7	18	4.3
Marital status										
Never married	279	92.4	12	4.0	11	3.6	302	94.4	18	5.6
Previously married	278	93.6	7	2.4	12	4.0	297	95.8	13	4.2
Age in years*										
40–49	234	93.2	3	1.2	14	5.6	251	93.3	18	6.7
50–59	172	91.0	10	5.3	7	3.7	189	95.9	8	4.1
60–69	114	95.8	3	2.5	2	1.7	119	96.7	4	3.3
70–75	37	92.5	3	7.5	0	0.0	40	97.6	1	2.4
Level of formal education										
High school, some college, technical training	230	97.5	6	2.5	NA		236	92.5	19	7.5
College degree or more	324	96.1	13	3.9			337	97.1	10	2.9
Working full-time or part-time*										
No	157	94.0	10	6.0	NA		167	94.9	9	5.1
Yes	393	97.8	9	2.2			402	95.3	20	4.7
Hispanic ethnicity ^†^										
Yes	21	91.3	2	8.7	NA		23	85.2	4	14.8
No	530	97.1	16	2.9			546	95.6	25	4.4
Race										
Black, Native American, Biracial, Multiracial	128	94.8	7	5.2	NA		135	93.1	10	6.9
White	422	97.5	11	2.5			433	96.0	18	4.0
Source of recruitment										
Print media	124	95.4	2	1.5	4	3.1	130	97.7	3	2.3
Community settings	91	93.8	2	2.1	4	4.1	97	94.2	6	5.8
Mailings/flyers	104	94.6	2	1.8	4	3.6	110	95.7	5	4.4
Personal networks	118	90.8	9	6.9	3	2.5	130	94.9	7	5.1
Health fair/other	120	90.9	4	3.0	8	6.1	132	93.0	10	7.0

WPW = Women who partner with women; WPM = Women who partner with men; NPP = No partner preference.

NA = Not available.

* Significant difference across type of participation for subjects agreeing to randomization

^† ^Significant difference between randomized and self-choice conditions.

### Indicators of cancer screening by randomly assigned data collection mode

Overall, there were few significant differences and no definitive patterns from the analyses of the self-reported screening variables by mode of data collection (Table 4). Compared to CATI, women assigned to SAMQ were half as likely to report two or more years between most recent mammograms and to report ever changing the place they went for a cancer screening because of embarrassment showing their body to a health care provider. Women assigned to CASI were less likely to report being overdue for Pap testing (no Pap test in past three years) and were less likely to report that Pap testing was difficult due to body shape or size.

**Table 4:** Self-reported cancer screening behaviours by interview mode (n = 557)*, Rhode Island, 2003–2005

Response	**CATI (n = 194)**		**SAMQ (n = 193)**		**CASI (n = 170)**	
	**%**	**AOR (95% CI)**	**%**	**AOR (95% CI)**	**%**	**AOR (95% CI)**
*Mammography*						
No mammogram in past 2 years	16.5	reference	14.0	0.8 (0.5 – 1.5)	11.8	0.5 (0.3 – 1.0)
Ever put off or avoided mammography	41.2	reference	41.5	1.0 (0.6 – 1.5)	32.4	0.7 (0.4 – 1.0)
2 or more years between most recent mammograms	23.2	reference	11.9	0.5 (0.3 – 0.8)	17.7	0.7 (0.4 – 1.2)
No plan to get a mammogram within next 2 years	11.9	reference	12.4	1.1 (0.6 – 2.1)	15.9	1.4 (0.8 – 2.7)
Mammography difficult due to shape or size of breasts	51.0	reference	49.7	0.9 (0.6 – 1.4)	45.9	0.8 (0.5 – 1.2)
*Pap testing*				'		
No Pap test in past 3 years	14.9	reference	14.5	1.0 (0.6 – 1.8)	10.0	0.5 (0.2 – 0.9)
Ever put off or avoided Pap testing	33.0	reference	41.5	1.4 (0.9 – 2.2)	31.8	1.0 (0.6 – 1.5)
3 or more years between most recent Pap tests	11.9	reference	14.5	1.4 (0.7 – 2.5)	10.6	0.9 (0.4 – 1.7)
No plan to get a Pap test within the next 3 years	23.2	reference	20.2	0.8 (0.5 – 1.4)	21.8	0.9 (0.5 – 1.4)
Pap testing difficult due to body shape or size	33.0	reference	34.2	1.0 (0.7 – 1.6)	19.4	0.5 (0.3 – 0.8)
*General Cancer Screening*						
Put off or avoided cancer screening due to problems with work schedules, transportation, health limitations, or dependent care	28.4	reference	31.6	1.2 (0.8 – 1.9)	20.0	0.7 (0.4 – 1.1)
Put off or avoided cancer screening because embarrassed to show body	17.5	reference	17.6	1.0 (0.6 – 1.8)	16.5	1.0 (0.6 – 1.8)
Changed place of cancer screening because embarrassed to show body	13.9	reference	7.3	0.4 (0.2 – 0.9)	11.8	0.8 (0.4 – 1.5)

*All models adjusted for age, education, income, race, marital status, partner gender, and source of recruitment.

SAMQ = Self-Administered Mailed Questionnaire; CATI = Computer Assisted Telephone Interview; CASI = Computer Assisted Self Interview

When using SAMQ as the reference (not shown in Table 4), women assigned to CASI were less likely to report difficulties with Pap tests due to body shape or size (AOR = 0.5, 95% CI = 0.3 – 0.7) and less likely to report any barriers to cancer screenings (AOR = 0.5, 95% CI = 0.3 – 0.9; analyses available upon request).

We tested for interactions between partner gender and mode of interview. There were only two significant interactions. WPW were less likely to report two or more years between most recent mammograms in CASI and SAMQ than CATI (CASI: AOR = 0.3, 95% CI = 0.1 – 0.8; SAMQ: AOR = 0.4, 95% CI = 0.1 – 0.9). In addition, WPW were less likely to report difficulty with Pap testing due to body shape or size in CASI and SAMQ than in CATI (CASI: AOR = 0.3, 95% CI = 0.1 – 0.7; SAMQ: AOR = 0.6, 95% CI = 0.3 – 1.2).

### Indicators of cancer screening by type of CASI condition

We replicated the results by separating the type of CASI condition (CASI-I vs. CASI-D) using CATI as the reference [analyses available upon request]. Compared to CATI, women completing CASI-I were more likely to report no plan to get a mammogram within the next two years (22.2% vs. 11.9%, AOR = 2.1, 95% CI = 1.0 – 4.6). On the other hand, women completing CASI-D were less likely to report being overdue for Pap testing (no Pap test in past 3 years; 5.1% vs. 15.0%, AOR = 0.2, 95% CI = 0.0 – 0.6) and less likely to report no plan to get a Pap test within the next three years (12.2% vs. 23.2%, AOR = 0.5, 95% CI = 0.2 – 0.9). Similar to CASI overall, women in CASI-D were less likely to report Pap testing being difficult due to body shape or size compared to those assigned to CATI (17.4% vs. 33.0%, AOR = 0.4, 95% CI = 0.2 – 0.8). Women in CASI-D were also less likely to report any barriers to cancer screening (14.3% vs. 28.4%, AOR = 0.5, 95% CI = 0.2 – 0.9).

Compared to SAMQ, women completing CASI-D were less likely to report: being overdue for Pap testing (5.1% vs. 14.5%, AOR = 0.2, 95% CI = 0.0 – 0.6), ever putting off a Pap test (26.5% vs. 41.5%, AOR = 0.5, 95% CI = 0.3 – 0.9), Pap testing being difficult due to body size or shape (17.4% vs. 34.2%, AOR = 0.4, 95% CI = 0.2 – 0.7), and barriers to cancer screening (14.3% vs. 31.6%, AOR = 0.4, 95% CI = 0.2 – 0.7).

Finally, we replicated all the analyses after removing the 23 women who refused to select a partner gender. The results were not significantly different.

## Discussion

Our findings contribute preliminary evidence of the effect of interview mode on responses to indicators of cancer screening behaviours among middle-aged and older heterosexual and sexual minority women. These findings add to the body of research about methods that can be used to best identify subgroups of the population most at risk for not receiving recommended cancer screenings. Women were randomly assigned to one of three data collection methods: computer assisted telephone interview (CATI), self-administered mailed questionnaire (SAMQ) and computer-assisted self-interview (CASI). Women assigned to CASI could choose to complete the assessment during an in-person CASI (CASI-I) or by receiving the questionnaire on disk (CASI-D).

We examined the effects of randomized interview mode on responses to items associated with mammography and Pap test screening. Overall, we found few meaningful differences by mode of data collection for indicators of cancer screening. Surprisingly, among the few significant mode differences, we found that women who were interviewed by research staff (CATI) were more likely than those not interviewed (CASI, SAMQ) to have an unfavourable status on the indicators. Women in the CATI mode were more likely to report being off-schedule for recent Pap testing than women in CASI and the trend was similar, but non-significant, for mammography. The other significant findings associated with cancer screening behaviours were between CASI conditions. Because we did not randomize women into the different computer-assisted methods, we cannot rule out selection bias as a threat to the validity of the findings. Furthermore, given the lower response rate in the CASI condition (Figure 1), apparently higher rates of recent screening among women in CASI may be due to the fact that those who completed the assessment were also those most knowledgeable about cancer screening recommendations. Therefore, we cannot conclude that any mode of data collection has a consistent effect on rates of reporting screening behaviours.

There are potential reasons why we did not find consistent mode differences in our sample. First, items about cancer screenings may not be considered sensitive or associated with social rejection since questions about mammography and Pap testing are routinely asked of women 40–75 years in clinical settings. Second, many of the studies that showed differences between CASI and other modes of data collection were conducted and published in the early and late 1990s [11,14-16,36,37]. At that time, CASI was a novel interview mode. The increased access to, and use of, computers may explain why we did not find more significant differences between CASI and the other data collection modes. Finally, due to the relatively high percentages of women reporting mammography and Pap testing at recommended intervals (more than 80% for both behaviours), we may not have had sufficient power to detect statistically significant differences. With a sample size of 364 for comparisons between CATI and CASI, we only had statistical power of 0.78 to detect differences in means of 0.10 or higher with a standard deviation of 0.35. Similarly, we only had statistical power of 0.80 to detect comparable differences in means of 0.10 or higher between CATI and SAMQ with a sample size of 387. However, because the percentages of endorsement were remarkably consistent across mode for several items, it is not likely that increased sample sizes would change the conclusions substantially.

Another study objective was to determine whether the effects of interview mode differed by partner gender. We found only two significant mode differences for items related to self-reported mammography and Pap test screening by partner gender. There are several potential reasons that may explain the lack of more significant findings. First, Rhode Island is one of only a few states in the United States to have non-discriminatory policies towards sexual minorities. Therefore, within the political and social context, women in Rhode Island may be more willing than women in other parts of the country to disclose potentially unfavourable information. Second, all women interested in study participation were required to answer screening questions about marital status and partner gender prior to study enrollment. Asking these screening items provided women with examples of the types of questions that would be asked in the study. Women who considered these items too personal may have declined study participation. Finally, sample size, particularly for sexual minority women, may have limited our ability to detect important mode differences.

In the CASI condition, WPW were significantly more likely to select the mailed computer disk than WPM/NPP when given a choice of completing the assessment by a laptop provided by the research team or by a disk mailed to the participant's home. WPW were also more likely to have a college education, be employed full or part-time, have higher incomes and identify as White than WPM/NPP. Therefore, it is likely that WPW had greater access to, and experience with, computers than WPM/NPP and were able to complete the assessment independent of assistance from a research assistant with a laptop computer. Given our findings, we encourage future studies to further explore women's preferences for data collection methods and whether mode of data collection influences the responses of middle-aged and older sexual minorities.

Our findings also provide information about the feasibility of different methods for collecting data from a traditionally under-represented group of women. Of the 630 women who were eligible and enrolled in the study, 95% agreed to be randomized to one of three modes of data collection. Not surprisingly, women who were more likely to have access to a computer (e.g., more education, employed, white race) chose CASI-D. Women who refused randomization (self-choice) were more likely to have less than a college degree, to identify as Hispanic, and to choose SAMQ. Despite the informed consent process, women in the self-choice option may not have completely understood the concept of randomization and been concerned about the implications of agreeing to randomization. They may have chosen the mode that was most familiar to them, offered the most perceived anonymity, and provided the greatest degree of flexibility in completing the assessment (e.g., time and availability of assistance with question understanding).

We obtained an overall response rate of 93%. This response rate is generally higher than for most other studies, particularly SAMQs, and is a strength of our study because of the low potential for non-response bias. The high response rate is likely a result of the initial contact we had with women during recruitment and screening for eligibility. Unfortunately, we do not have data to inform other studies of comparable populations that do not employ similar pre-survey contact with participants.

Despite the high overall response rate, we found noteworthy differences in response rate by mode. The response rates for CATI and SAMQ were over 95%, while only 86% for CASI. The lower response rate by computer was not unexpected given other mode experiments [19] and the age of the participants. There were likely some women with less experience using computers who, despite initially agreeing to participate, worried about their ability to correctly use the software or feared unknown potential consequences of responding to a computer program. Additionally, women may have had technical difficulties with the computer that we were unaware of because they indicated that they were no longer interested in study participation rather than acknowledging problems with computer software.

We also found that more contact attempts with participants were required for CASI relative to SAMQ and CATI to achieve comparable response rates (Figure 2). Furthermore, the estimated costs per randomized participant were approximately $60 for CASI compared to $30 for SAMQ and $20 for CATI. Within the CASI condition, the cost per participant was about $115 for CASI-I and $20 for CASI-D. Had we used Internet-based data collection, the costs associated with CASI would have been substantially lower. However, the sample would have been biased towards women with higher socioeconomic positions who had access to a computer. Women in our sample who chose in-person CASI were more likely to identify as a racial minority, to be less educated and not employed compared to those who chose to complete the questionnaire on a disk that was mailed to them.

In addition to sample size, there are a number of other study limitations. First, to include sufficient numbers of sexual minorities, we used non-probability based sampling methods. Our sample was highly educated, predominantly white, and employed, with relatively higher incomes. Unfortunately, because sexual orientation is not asked of all individuals in the Census or on any large state-wide population-based survey, we do not have data to compare our sample to the eligible Rhode Island population. Therefore, care should be taken when generalizing our findings. We also did not use methods to verify self-reported data and cannot confirm whether there was substantial over- or under-reporting where differences were observed across modes. Finally, we cannot discern which mode provided the most accurate estimates of true behaviour, nor can we distinguish the extent to which differences across modes indicate differences in accuracy of reports as opposed to mode artefacts. However, given the few statistically significant differences, it appears that the incidence of mode artefacts is low.

## Conclusion

Using computer-assisted self-interviewing for surveillance and intervention studies may result in lower response rates than telephone interviewing or self-administered mailed questionnaires. However, there does not appear to be consistent differences by mode of data collection for responses to indicators of mammography and Pap test screening among middle-aged and older women who complete the assessment. Therefore, other measures such as efficiency and cost-effectiveness of the mode should also be considered when determining the most appropriate form of data collection for use in monitoring indicators of cancer detection and control.

## Competing interests

The author(s) declare that they have no competing interests.

## Authors' contributions

MAC was responsible for planning the research, leading the recruitment and data collection efforts, and writing the first draft of the article. MLR analyzed the data and contributed to the interpretation of study findings. GFA managed the study database and oversaw the day-to-day study operations. WR assisted in the conceptualization of the project and the interpretation of the results. FJK assisted in the interpretation of the data and presentation of the results. All authors actively contributed to writing and editing of the article.

## Pre-publication history

The pre-publication history for this paper can be accessed here:


